# Diagnosis of Malignant Tumors of the Upper Jaw in Youth

**Published:** 1880-10

**Authors:** L. Mclane Tiffany

**Affiliations:** Professor of Operative Surgery, University of Maryland


### THE DENTAL REGISTER.

VOL. XXXIV.] OCTOBER, 1880. [No. 10.

### DIAGNOSIS OF MALIGNANT TUMORS OF THE UPPER JAW IN YOUTH.

BY L. MC LANE TIFFANY, M. D., PROFESSOR OF OPERATIVE SURGERY, UNIVERSITY OF MARYLAND.

Malignant tumors of the upper jaw occurring in childhood, are characterized by extremely rapid increase at the expense of surrounding tissue. Removal, to be effective, should be complete and an early diagnosis is absolutely essential that the surgeon may offer to his patient reasonable hope of cure. With the laudable intention of assisting the diagnosis, the following cases are related and commented upon:

CASE 1.—A. S., of Harford County, aged 9 years, female, suffered some slight pain about the right upper canine tooth, not sufficient, however, to attract more than passing notice. Two weeks later a slight fullness appeared by the side of the tooth at the border of the gum; this increased, was considered to be "proud flesh" and touched with lunar caustic. The caustic failing to arrest growth the case was sent to me. I found a soft, vascular, bright red, tumor, partially surrounding the canine tooth, involving the gum, and manifestly growing from the alveolar border of the jaw. Handling caused some bleeding, increase of the tumor in size was said to be rapid. A diagnosis of sarcoma was arrived at. Removal was effected by extracting the canine and two adjacent teeth then cutting away the gum and alveolar border with forceps. Hemorrhage was slight and the patient returned home in a week.

Three weeks later A. S., again consulted me.

Protruding from the upper jaw in the gap left by the former operation was a red tumor adherent by a broad base. The growth was about the third of an inch in diameter, the progress of three weeks; evidently the first operation had not reached the spot from which the tumor originated. Unwilling to remove the upper jaw and mindful of the fact that the upper jaw at the age stated has a broad alveolar border, I operated as follows through the mouth, so as to cause no scar, a matter of prime importance in the female; the cheek and lip were freely dissected from the bone as far up as the infra orbital foramen. all teeth posterior to the lateral incisor were extracted, and then with strong forceps having blades set at angle to the handles the whole alveolar border was removed, the line of section running from above downwards and without inwards. The line of section was so directed as just not to open the nasal cavity. Chloride of zinc pure, was rubbed into the exposed bone surface.

To avoid the inconvenience which would result from the passage of blood into the trachea, as well as the danger, the operation was done with the patient resting on the belly, the head raised facing a window, supported of course by assistants. The lateral light illumined the mouth well, and the blood ran at once from the mouth to the floor, not passing backwards, which indeed would have been up hill. This device of position rendered the operation easy and simple. Some little hemorrhage occurred during the afternoon, which however, was arrested without much trouble, after which all went well. After treatment consisted of mouth-washes and tonics, the wound healed over rapidly and no deformity was present. A set of teeth will be adjusted at the proper time. It is now five and a half years since the operation was performed and recurrence has not taken place. Microscopic examination showed the tumor to consist of round and spindle cells, vessels, delicate connective tissue.

Case 2.—W. O., male, from Cecil County, aged 18 months, was sent to me, with a history extending over one month only. A lump was first noticed at the free border of the gum corresponding to the first molar; the lump grew rapidly and bled on handling. Nitrate of silver had not arrested progress. Examination showed a soft, bright red, moderately formed tumor the size of a pea, attached to the alveolar border of the jaw by a broad base. Removal was effected as in case No. 1. The same position facing a window was made use of with equally happy effect. Two years later when heard from, recurrence of the growth had not been observed.

Case 3.—I. B., aged 16 years, of Baltimore County, female, noticed a swelling of the left upper jaw, and attributed it to a tooth. Enlargement increased, remaining hard however, an exploratory incision was made giving exit to blood only. I. B. then had a tooth extracted, but failed to diminish the size of her jaw. Four months after the commencement of her trouble she was referred to me by her attending physician. I. B. was a large, well formed, healthy girl, functions said to be regular. The left upper jaw was the seat of a tumor partly covered by bone, partly not, firm, elastic, extending from the middle line in front to the last molar tooth not encroaching upon the nostril, not involving the hard palate beyond the middle line. A finger passed up behind the soft palate showed the growth in that direction to be limited by the maxilla. While the tumor distended the cheek, the skin was not involved. The eye was not protruded, although the swelling extended to the floor of the orbit. Growth during the last six weeks very rapid. Diagnosis, Sarcoma. Treatment, removal of the upper jaw with tumor. This was effected on January 7th of this year; chloroform as the anæsthetic, for the use of Paquelin's cautery was intended. Laryngo-Tracheotomy was effected, the incisions being made with the gas cautery, because the patient has a vascular goitre encroaching upon the crico-thyroid space; notwithstanding the knife was used at red heat and slowly, a large vein required ligature. A tube being inserted in the wind-pipe anæsthesia was kept up through it, and the operator thus gained undisturbed possession of the mouth. The pharynx and mouth were stuffed with sponges. An incision was carried through the lower lid, commencing just external to the puncture downwards by the nose, around the ala and through the middle line of the lip, thus forming with lid and lip a large quadrangular flap. This flap was reflected so as to thoroughly uncover the jaw tumor, the central left incisor was extracted, the alveolar border and hard palate were divided with saw and forceps, the soft palate was divided by the cautery. The nose was separated from the left jaw and turned to the right. The eye was raised with soft parts from the floor of the orbit and held up, one blade of cutting forceps was put in the orbit, one in the nose, and the intervening bone cut through. The malar-maxillary joining was divided with saw and forceps, the jaw was then grasped with strong pliers and twisted from its bed. But little hemorrhage occurred, the cheek flap giving most blood and requiring a touch or two with the cautery. Collapse came on suddenly, necessitating lowering the head and hypodermic injections of whiskey. The patient reacting, was put to bed with hot bottles. Wound was united by suture. Recovery was retarded by a slight facial erysipelas which caused the lower lid to unite with a small depression, abscesses formed at the site of several hypodermic punctures in the limbs. The cavity left by removal of tumor was syringed several times daily, and the cheek supported by a pad of oiled oakum.

At present time patient is quite well, a slight V in the lower lid shows where the erysipelas retarded union; this can be easly corrected, however. The soft palate has been preserved. Microscopic examination showed the tumor to be composed of spindle cells with but scant intercellular tissue and many vessels. A spindle cell sarcoma; no myeloid cells found.

No gladular affection was present. The cases related have certain points in common worthy perhaps of notice. The patients were all inhabitants of the country, and in robust health. I am not aware of any observations tending to show that town-people are in any way exempt from jaw tumors, more than the extra urban population. Age, rapidity of growth, absence of pain, locality affected, were common to all, and indicate, of course, sarcoma, confirmatory evidence of the same being supplied by microscopic examination of the removed growths.

The disease in cases one and two probably started from the periosteum lining a tooth socket, and growing in the direction of least resistance, first appeared between the tooth and the gum, increasing rapidly. This would correspond to the clinical life of a periosteal sarcoma, which is no sooner freed from pressure than it sprouts quickly and dispenses with the bony plates in which it clothes its early increase; indeed, sarcomata, periosteal or not, prejecting into a cavity grow rapidly and become pedunculated.

Polyp of the dental pulp, as described by Tomes, and exuberant granulations protruding by the side of a decayed tooth, present a certain resemblance to the first appearances noticed in cases one and two, but the absence of all pain, throbbing, œdema or decay, and the failure of nitrate of silver to arrest progress, led at once to the proper diagnosis and institution of treatment. Case three, however, was of a more serious nature, the morbid growth commencing probably in the body of the jaw, showing no tendency to appear by the side of a tooth but increasing in the direction of the cheek as well as towards the mouth; a typical case of the severest form of jaw tumor. Sarcoma of the upper jaw is the privilege of youth, rarely being observed after the 24th year. A cause is generally wanting, and while nasopharyngeal polyps are more often found in males, the growth in question affects the two sexes about equally.

Progress is rapid, and increase takes place in the direction of least resistance, namely, towards the cheek and mouth. Protrusion of the eye from pressure upwards upon the floor of the orbit, and interference with the passage of air through the nostril are symptoms of very late occurrence, and indicate a growth within the antrum, or from the base of the skull, rather than from the body of the bone. Invasion is insidious, attention being attracted by increased size at the affected locality more often than by pain or loss of function, both of which symptoms are absent until a late stage of the disease.

Increase of tumor while surrounded by bone is usually not very fast; the bone being perforated, rapid increase occurs with pedunculation, but without hemorrhage, if upon a mucus surface; no pedunculation, but always hemorrhage upon a skin surface, a fact of some clinical interest. Glandular infection wanting always. The shape of the tumor is defined by the jaw with a general tendency towards knobby and rounded outgrowths. A thermometer will show the temperature of the growth to be two or three degrees (2° or 3° F.) higher than the corresponding point on the opposite side of the body a fact, inflammatory symptoms wanting, which has ominous clinical importance. This is true, whether the temperature be taken on the skin surface or in the mouth. Estlander of Helsingfors, Finland, in 1878, called attention to elevated temperature in Osteo Sarcoma, and this I have confirmed in several instances. When not covered by bone the growth imparts to the fingers of the examiner a sensation as of a tense elastic body, with perhaps false fluctuation enough to deceive even the most wary; especially do rounded outgrowths present this peculiarity.

Cystic tumors of the upper jaw sometimes expands the bone until it is of parchment thinness, and give a sensation as of crackling when handled and pressed. I am not aware that solid malignant tumors present this symptom ever. No part of the body is more accessible for diagnostic purposes than the upper jaw, the natural openings of mouth, nose and orbit afford means of explorations in three directions, while a finger passed up behind the soft palate can reach the base of the skull and thus examine the posterior surface of the bone. The remaining bone surface, its condition, can be appreciated through the cheek. Round and spindle celled sarcomata increase with rapidity, the presence of myeloid cells (myeloplaxes) indicates slower growth. An incision into the tumor is sometimes required in order to verify the diagnosis, it is always proper before operation. From sarcoma dental abscess and suppuration in the antrum are distinguished by symptoms of inflammatory actions, also incision yields pus.

A dentigerous cyst occurs during the period of second dentition, forms about a misplaced tooth which is wanting in its proper situation, approaches the surface and gives to the fingers a sensation of cracking, is rarely of large size; incision yields thick, clear fluid, and discloses the missing tooth. Diagnosis of dropsy or of polyp of the antrum, is never absolute without incision. The different varieties of odontoma as described by Broca offer no difficulties of diagnosis.

Prognosis. Very simple, progressive increase and death by repeated hemorrhages, or by interfering with necessary functions, *i. e.* respiration or deglutition. Treatment consists in removing the tumor, together with the bone from which it grows, otherwise there is local recurrence, and it is better to cut freely where there is doubt as to the amount of bone involved rather than to leave tissue which may become the nidus of future reproduction.

Reflecting the lip and cheek if tumor is small, and cutting as in cases one and two, in view of the cure affected seems worthy of commendation. By this method a large amount of bone can be removed through the mouth without cutting the face. The position also in which the patients were placed, with the head somewhat raised, facing a window, gave a good light and obviated all danger of blood entering the air passages, thus removing one anxiety from the mind of the operator.

Case 3 required a more serious procedure than either of the others. The usual skin incision was not adopted, but the lower lid was divided and the opening between the lids utilized instead, carrying an incision across the face below the orbit. By this means an additional scar upon the face was avoided, and the large quadrilateral flap containing the facial nerve and artery uninjured, being reflected, thoroughly exposed the jaw.

The performance of tracheotomy prior to removing an upper jaw is not usually considered necessary, but the comfort to an operator of knowing that respiration will not be interrupted by flow of blood, and of having entire control of the head, can scarcely be overestimated, and greatly outweighs any possible risk from the additional neck wound. The crico-thyroid membrane was opened with the thermo-cautery and was not attended by the usual cough and splutter which follows the ordinary method by bistoury. There was no accelerated respiration or struggle, but breathing went on undisturbed through the neck. To me the experience was most singular, and I shall not fail to repeat it when opportunity presents.

I am not aware that tracheotomy with Paquelin's cautery has been often done in this country, if at all. While the first case of upper jaw removal, successful, is credited to Jamison, of Baltimore, in the early part of the century, it does not appear to have been repeated in this city until 1875, when it was by Christopher Johnston, M. D. The patient was exhibited at the Clinical Society 2 years after the operation; the deformity was very slight. In that case Diffenbach's line of incision was followed.

